# The C1431T polymorphism of peroxisome proliferator activated receptor γ (PPARγ) is associated with low risk of diabetes in a Pakistani cohort

**DOI:** 10.1186/s13098-016-0183-z

**Published:** 2016-09-13

**Authors:** Huma Butt, Shabana, Shahida Hasnain

**Affiliations:** 1 Department of Microbiology and Molecular Genetics, University of the Punjab, Lahore, Pakistan; 2 The Women University Multan, Multan, Pakistan

**Keywords:** Diabetes, C1431T, Peroxisome proliferators-activated receptor, Pakistan

## Abstract

**Background:**

Diabetes is a socioeconomic burden in Pakistan. International diabetes federation reported 6.9 million cases of diabetes and 87,548 deaths due to diabetes in Pakistan in 2014. Peroxisome proliferators-activated receptors are transcription factors, regulating several physiological processes.

**Aim:**

The aim of the current study was to determine the prevalence of silent variant C1431T in exon 6 of PPAR-y and analyze its effect on various anthropometric and biochemical parameters in a Pakistani cohort.

**Methods:**

We collected 926 samples, 500 healthy controls (fasting blood sugar <99 mg/dL, random blood sugar <126 mg/dL) and 426 cases with diabetes (fasting blood sugar >99 mg/dL, random blood sugar >126 mg/dL). The genotyping was done by polymerase chain reaction restriction fragment length polymorphism (PCR–RFLP) and serum biochemical parameters were determined by commercially available kits.

**Results:**

The genotyping results by RLFP showed allelic frequency C = 61.2 % and T = 38.8 % in controls while C = 74.5 % and T = 25.5 % in cases (OR 0.536, CI 0.439–0.655, *p* = 8.2 × 10^−10^) and genotypic frequency CC = 38.8 %, CT = 44.7 %, TT = 16.5 % in controls. While CC = 53.6 %, CT = 41.4 %, TT = 5.1 % in cases (OR 0.544, CI 0.408–0.726, *p* = 2.3 × 10^−10^). The rare T allele appeared to be a protective allele i.e., the presence of rare allele lowered the risk of diabetes in the studied cohort. The biochemical and anthropometric parameters were analyzed for any significant association with the SNP showing that C1431T variant has an association with BMI, weight, fasting glucose and LDLC. However, no significant association was found with age, gender, height, HDLC, TC, triglycerides and leptin.

**Conclusion:**

In conclusion, the presence of minor allele lowers the risk of diabetes and the effect may involve modulating certain serum parameters.

## Background

Diabetes is a metabolic disorder, associated with a number of specific and non-specific micro/macrovascular complications. It is characterized by hyperglycemia, which can either be due to improper functioning of the pancreatic β-cells which decreases insulin production, or the resistance of body cells to insulin wherein insulin production by β-cells is normal but cells cannot respond to it [1].

Diabetes is a common cause of premature mortality and prolonged illness in Pakistan. It is highly prevalent in almost all regions of Pakistan, with an overall prevalence of 22.04 % in urban and 17.15 % in rural areas. The gender wise distribution of diabetes in different provinces of Pakistan is: Punjab; males 16.6 %, females 19.3 %, Khyber Pakhtunkhwa; 11.1 % both sexes, Balochistan; 10.8 % both sexes, Sindh; males 16.2 %, females 11.7 % [2].

Peroxisome proliferators-activated receptors (PPARs) are transcription factors and members of the super family of steroid hormone receptors, activated by lipid soluble membrane permeable ligands, regulating the expression of several target genes. The physiological processes affected include insulin signaling, lipid and glucose metabolism and adipogenesis [3]. There are three types of PPARs, PPARα, PPARβ and PPARƳ among which variations in PPARƳ gene are mostly implicated in obesity, diabetes and many other disorders [4].

PPARγ is located on chromosome 3p25 [5]. The functions of this nuclear transcription factor include regulation of multiple genes involved in energy regulation, glucose and lipid metabolism, and plays role in insulin sensitivity. A silent variant present in the exon 6 of the PPARγ gene involves substitution of cysteine at nucleotide position 1431 with thiamine. It was identified in 1998 by Meirhaeghe et al. and is also known as His477His. It has been shown to be associated with various diseases and extensive studies have been carried out to assess the effects of this polymorphism on many aspects of human physiology [6]. This variant has been investigated in different populations but the association of this variant with obesity, diabetes and cardiac disorders as a protective SNP remains controversial [7–11]. The aim of the study was to check the association pattern of PPARƳ C1431T polymorphism with diabetes in the diabetic patients and healthy controls of Pakistani origin, and determine its effects on selected anthropometric and serum biochemical traits.

## Methods

### Subject recruitment

A total of 926 samples (426 clinically diagnosed type 2 diabetic cases and 500 normal controls) were recruited from the hospitals and diabetic centers of Punjab, Pakistan. Inclusion criteria for diabetic subjects were (i) diabetes diagnosed according to etiologic classification of diabetes by the International Diabetes Federation (IDF) and (ii) confirmation that all the grandparents of the subjects are of Pakistani origin. The exclusion criteria consisted were the presence of any infectious disease, conditions where phlebotomy is contra-indicated, age below 10 years, body mass index (BMI) ≤18.5 kg/m^2^, pregnancy, handicapped/mentally disturbed individuals, obese (obesity defined as: BMI >23 kg/m^2^ as overweight and BMI> 26 kg/m^2^ as obese, criteria used for Asian populations as described previously [12]), cancer and ethnicity other than Pakistani. The recruitment was done between July 2014 and April 2015. The controls were healthy subjects from the general population with normal blood sugar levels (<126 mg/dL random or <99 mg/dL fasting). All the subjects were genetically unrelated and gave written informed consent. The procedures employed were according to the Helsinki declaration and an ethical approval was obtained from the institutional ethical board.

### Blood sampling

Venous blood was collected from the subjects in the fasting state. Five milliliters blood was collected from the median cubital vein using aseptic measures. Half was dispensed in an EDTA vial and used for DNA isolation while rest half was poured in a gel clot activator containing vial and used for biochemical analysis.

### Biochemical parameters’ determination

Gel clot activator containing vial was centrifuged to separate plasma for determination of biochemical parameters. Serum was screened for HBV, HCV and HIV. Serum fasting plasma glucose (FPG), total cholesterol (TC), triglycerides (TG), high density and low density lipoprotein cholesterol (HDL-c, LDL-c), were determined using commercially available kits (Spectrum Diagnostics, Egypt), leptin was measured by LDN Nordhorn leptin ELISA kit whereas insulin concentration was measured using an electrochemiluminescence method as described previously [13], and HOMA-IR was calculated.

### Genotyping

Genomic DNA isolation was done manually by salting out method and results were checked on 1 % agarose gel. The DNA was quantified by measuring the optical density and was standardized to a final concentration of 10 ng/µl. PCR was done with the following set of primers. Forward primer 5′-CCTCCCCACCTATTTAAGATA-3 and reverse primer 5′-CGGGATCCGTACAAGTCCTTGTAGATCTCC-3′. PCR consisted of an initial denaturation at 95 °C for 2 min, followed by 35 cycles of denaturation at 95 °C for 30 s, annealing at 57 °C for 45 s, extension at 72 °C for 45 s and a final extension at 72 °C for 10 min. Advanced Primus 96 (PeqLab) thermal cycler was used for amplification. 397 bp PCR product was subjected to restriction digestion using restriction enzyme *Bsa*A1 at 30 °C for 6 h [14]. It cuts the product in wild type state producing fragments of 305 bp and 92 bp. PCR and digestion products were analyzed on 2 % agarose gel.

## Results

The diabetic cases studied, were from the government hospitals, the diabetic centers and laboratories while controls were unrelated subjects, but ethnically matched and healthy individuals from the general population. The general characteristics of the subjects are given in Table 1 and show that, except for height and gender (*p* value 0.314 and 0.108 respectively), all other parameters were found to be significantly different between cases and controls. The mean age (years) of cases was 42.12 ± 9.53 and of controls was 49.84 ± 8.46 whereas for males, it was 47 ± 12.7 and of females was 46 ± 11.6. The proportion of diabetic patients suffering population from other disorders, including foot ulcer, cardiovascular disease, hypocholesteremia, hypertension, nephropathy, and retinopathy was higher in the cases than the controls (Table 2). The average blood pressure of the diabetic patients was normal 120/80 mmHg^−1^ however the patients suffering from cardiovascular disease and nephropathy showed high blood pressure 140/80 mmHg^−1^.

**Table 1:** General characteristics of the study subjects

Parameters	Diabetic (n = 426)	Non-diabetic (n = 500)	*p* value
Gender			
Male	228	272	0.108
Female	198	228	0.108
Age (yr)	47.55 ± 12.3	35.78 ± 13.4	0.0001*
Height (ft)	5.50 ± 0.3	5.49 ± 0.59	0.756
Weight (Kg)	68.6 ± 13.83	65.38 ± 13.7	0.0004*
BMI (Kg/m^2^)	22.7 ± 5.6	21.67 ± 5.3	0.0043*

*BMI* body mass index, *n* total number

* Indicates significant differences

**Table 2:** Prevalence of comorbidities

Disorders	Frequency (%)	
	Cases (n = 426) (%)	Controls (n = 500)
Cardiovascular disease	8.1	2 %
Nephropathy	7	0
Retinopathy	3.4	0
Foot ulcer	3.8	0
Hypocholesteremia	7	1 %
Hypertension	8.9	2.3 %

The distribution of observed genotypes is given in Table 3. The study population was in Hardy–Weinberg equilibrium (*p* = 0.561). The allelic frequency was C = 0.745 and T = 0.255 in diabetic cases and C = 0.612 and T = 0.388 in the controls (OR 0.536, CI 0.439–0.655, *p* = 8.2 × 10^−10^), whereas genotypic frequency was CC = 0.536, CT = 0.414, and TT = 0.051 in cases and CC = 0.388, CT = 0.447, TT = 0.165 in controls (OR 0.544, CI 0.408–0.726, *p* = 2.3 × 10^−10^). Logistic regression was used to analyze the association of C1431T polymorphism with diabetes. The result showed that the polymorphism is significantly associated (*p* = 0.004) with diabetes in the Pakistani study subjects.

**Table 3:** Allelic and genotypic frequency in study subjects

Allele/genotype	Non-diabetic control (n = 500) (%)	Diabetic cases (n = 426) (%)	Odds ratio	95 % CI	*p* value
C	61.2	74.5	0.536	0.439–0.655	8.2 × 10^−10^
T	38.8	25.5			
CC	38.8	53.5	0.544	0.408–0.726	2.3 × 10^−10^
CT	44.7	41.4			
TT	16.5	5.1			

*CI* confidence interval

The serum was subjected to analysis of certain parameters reported to play role in progression to diabetes. There was a slight difference between the values of serum traits between the males and females. The association of variant C1431T with anthropometric traits (gender, age, height, weight, BMI) was analyzed by one way ANOVA (analysis of variance) and the results were confirmed by an independent *t*-test. The mean and the standard deviation values of the genotype TT were slightly less than the CC and CT genotype. The BMI (*p* = 2.1 × 10^−4^) and weight (*p* = 2.2 × 10^−5^) were significantly associated with the variant, however, age (*p* = 0.710) and height (*p* = 0.562) did not show association with C1431T polymorphism (p = 0.0761) (Table 4). Among biochemical parameters, the C1431T was significantly associated with the fasting glucose level (*p* = 2.1 × 10^−3^) and LDLC (*p* = 0.041). However, it showed no significant association with triglycerides (*p* = 0.862), total cholesterol (*p* = 0.809), HDLC (*p* = 0.775) and leptin (*p* = 0.701) (Table 5).

**Table 4:** Association between polymorphism of C1431T and anthropometric traits in study population

Anthropometric traits	Genotype	Mean ± SD	*p* value
Age (years)	CC	45.91 ± 13.04	0.710
	CT	32.15 ± 12.11	
	TT	47.83 ± 11.21	
Weight (Kg)	CC	78.20 ± 15.11	2.2 × 10^−5^*
	CT	72.98 ± 13.01	
	TT	63.54 ± 12.53	
Height (ft)	CC	5.41 ± 0.42	0.562
	CT	5.55 ± 0.26	
	TT	5.53 ± 0. 29	
BMI (Kg/m^2^)	CC	23.94 ± 3.28	2.1 × 10^−4^*
	CT	22.35 ± 5.01	
	TT	21.11 ± 4.52	

*BMI* body mass index

* Indicates significant association

**Table 5:** Association between C1431T polymorphism and serum traits

Serum traits	Genotype	Mean ± SD	*p* value
Glucose (mmol/L)	CC	7.11 ± 1.60	2.1 × 10^−3^*
	CT	6.20 ± 1.81	
	TT	5.22 ± 0.91	
Triglyceride (mmol/L)	CC	2.37 ± 0.86	0.862
	CT	2.37 ± 0.82	
	TT	2.30 ± 0.65	
Cholesterol (mmol/L)	CC	5.09 ± 0.99	0.809
	CT	5.07 ± 1.28	
	TT	4.97 ± 1.31	
LDLC (mmol/L)	CC	2.69 ± 0.71	0.041*
	CT	2.59 ± 0.65	
	TT	2.39 ± 0.45	
HDLC (mmol/L)	CC	1.40 ± 0.45	0.775
	CT	1.43 ± 0.47	
	TT	1.43 ± 0.41	
Leptin (mg/dL)	CC	3.59 ± 1.46	0.151
	CT	3.46 ± 0.74	
	TT	3.27 ± 1.11	

*HDLC* high density lipoprotein cholesterol; *LDLC* low density lipoprotein cholesterol

*Indicates significant association

## Discussion

The current study analyzed the allele and genotype frequencies of PPARγ gene C1431T polymorphism that is well studied in the Caucasians but no report on this variant is available for the Pakistani population. We aimed to find out its association, if any, with serum biochemical parameters in order to check a possible mechanism of action. This polymorphism is widely studied in different populations, but the results of its association patterns are controversial. Some studies have reported its protective role in diabetes, whereas others have found no such effect [15].

The mean weight observed in cases was higher than the normal population. In type 2 diabetes, the patient may eat more, but still feel hungry and results in overweight, whereas in type 1 diabetes patients rapid weight loss is observed [16]. In people with type 1 diabetes, insufficient insulin prevents the body from getting glucose from the blood into the body’s cells to use as energy. When this occurs, the body starts burning fat and muscle for energy, causing a reduction in overall body weight. Unexpected weight loss has often noticed in people prior to a diagnosis of type 1 diabetes.

There was a difference observed between the values of serum traits between the males and females, which may be due to the difference in certain sex-limited hormones, which are gender associated and have an influence on the body of individual metabolic processes. All the serum traits (total cholesterol, leptin, triglyceride, HDLC, LDLC and fasting glucose) showed increased values in diabetic patients as compared to non-diabetic controls. In previous studies it has been reported that the decreased insulin action, insulin deficiency and/or insulin resistance in tissues is the primary cause of diabetes. The most important defect in insulin-deficient subjects appears to be a deficiency of lipoprotein lipase, which is responsible for the removal of the triglyceride-rich lipoproteins. Cholesterol levels may be elevated and it is important to distinguish between LDL and HDL as the causes for these increases. HDLC level may increase in insulin-dependent subjects, whereas it may be decreased in obese non-insulin-dependent patients. Mild elevations of LDLC may occur in inadequately controlled type I and II diabetic patients [17].

Various studies have been conducted in other populations including Iranian [18], Mexican-Americans [19], African-Americans [20], Asian [21], and Chinese Han [22] populations regarding the role of PPARy variant C1431T to determine the allele frequency and the genotypic distribution. There have been no studies of the distribution of C1431T polymorphism in the Pakistani population in context to diabetes previously. The results were in accordance with some of the previous reports which showed that the polymorphism C1431T was associated with lower diabetes risks [23].

A highly showed significant association of C1431T polymorphism with BMI and weight was observed in the current study indicating a lowering effect of T allele on weight and BMI in the study subjects. These results are in accordance with a previous study conducted in Chinese and Quebec populations [11, 24]. The presence of the homozygous wild type appeared to increase BMI and weight, leading to obesity and an increased risk of having diabetes. The heterozygous study subjects showed a moderate trend of anthropometric traits. It has been reported in the previous studies that C1431T variant is associated with a higher BMI in type 2 diabetic patients [25].

Among the biochemical parameters, the only observed significant association of the polymorphism was with LDLC. The low density lipoprotein cholesterol serum concentration (LDL-C) is an independent diabetes and CHD risk factor and LDL-C lowering drugs reduce CHD risk [26, 27]. The biochemical and genetic basis of elevation in blood lipids is not fully understood, but their heritability has been estimated to be at least 50 % [28].

Single nucleotide polymorphisms (SNPs) can be used to examine whether a genetic biomarker is causally linked to a disease risk or not [29]. Most of the SNPs in the genes regulating blood lipids are inherited independently and affect lipid levels quantitatively [30]. In contrast to a large body of evidence available in Caucasians, data on the genetic regulation of lipids in Pakistani population is limited [31]. The allele frequencies of SNPs may show interethnic variations due to different linkage disequilibrium (LD) patterns, genetic drift, gene flow, mutation or admixture. Risk allele frequencies of some SNPs may be more prevalent in specific ethnic groups, or their effects may be modified due to environment or life style changes [32, 33]. This in turn may generate variations in disease outcomes across ethnicities [34]. Therefore, studies in non-European people will help to evaluate the true relevance of findings in European people [35, 36].

## Conclusion

In conclusion, these findings suggest that the presence of a polymorphism may not always increase the risk of a disease, rather sometimes a SNP may be beneficial, the C1431T polymorphism is an example of this. This SNP appeared to lower the risk of type 2 diabetes, the allele as well as genotype frequencies of minor allele were higher in controls as compared to cases. This variant showed significant association with weight, BMI and LDL-C indicating a potential role in the development of metabolic anomalies like diabetes.

## Abbreviations

WHO: World Health Organization
BMI: body mass index
WC: waist circumference
HC: hip circumference
FPG: fasting plasma glucose
GRS: genetic risk score
HDL-c: high density lipoprotein cholesterol
LDL-c: low density lipoprotein cholesterol
SBP: systolic blood pressure
DBP: diastolic blood pressure
HOMA-IR: homeostasis model assessment of insulin resistance

## References

[1] Kaul K, Tarr JM, Ahmad SI, Kohner EM, Chibber R (2013). Introduction to diabetes mellitus. Diabetes.

[2] Bahadar H, Mostafalou S, Abdollahi M (2014). Growing burden of diabetes in Pakistan and the possible role of arsenic and pesticides. J Diabetes Metab Disord.

[3] Youssef J, Badr MZ (2013). PPARs: history and advances. Peroxisome proliferator-activated receptors (PPARs).

[4] Black MH, Wu J, Takayanagi M, Wang N, Taylor KD, Haritunians T (2015). Variation in PPARG is associated with longitudinal change in insulin resistance in Mexican Americans at risk for type 2 diabetes. J Clin Endocrinol Metab.

[5] Greene M, Blumberg B, McBride O, Yi H, Kronquist K, Kwan K (1994). Isolation of the human peroxisome proliferator activated receptor gamma cDNA: expression in hematopoietic cells and chromosomal mapping. Gene Expr.

[6] Meirhaeghe A, Fajas L, Helbecque N, Cottel D, Lebel P, Dallongeville J (1998). A genetic polymorphism of the peroxisome proliferator-activated receptor γ gene influences plasma leptin levels in obese humans. Hum Mol Genet.

[7] Heude B, Pelloux V, Forhan A, Bedel J-F, Lacorte J-M, Clément K (2011). Association of the Pro12Ala and C1431T Variants of PPAR γ and Their Haplotypes with Susceptibility to Gestational Diabetes. J Clin Endocrinol Metab..

[8] Doney A, Fischer B, Cecil J, Boylan K, McGuigan F, Ralston S (2004). Association of the Pro12Ala and C1431T variants of PPARG and their haplotypes with susceptibility to type 2 diabetes. Diabetologia..

[9] Oladi M, Nohtani M, Avan A, Mirhafez SR, Tajbakhsh A, Ghasemi F (2015). Impact of the C1431T polymorphism of the peroxisome proliferator activated receptor-gamma (PPAR-γ) gene on fasted serum lipid levels in patients with coronary artery disease. Ann Nutr Metab.

[10] Prakash J, Srivastava N, Awasthi S, Agarwal C, Natu S, Rajpal N (2012). Association of PPAR-γ gene polymorphisms with obesity and obesity-associated phenotypes in north indian population. Am J Hum Biol..

[11] Zhou X, Chen J, Xu W (2012). Association between C1431T polymorphism in peroxisome proliferator-activated receptor-γ gene and coronary artery disease in Chinese Han population. Mol Biol Rep.

[12] Shabana, Shahid SU, Li KW, Acharya J, Cooper J, Hasnain S (2016). Effect of six type 2 diabetes susceptibility loci and an FTO variant on obesity in Pakistani subjects. Eur J Hum Genet..

[13] Shabana, Hasnain S (2015). Association of the leptin receptor Gln223Arg polymorphism with lipid profile in obese Pakistani subjects. Nutrition..

[14] Haseeb A, Iliyas M, Chakrabarti S, Farooqui AA, Naik SR, Ghosh S (2009). Single-nucleotide polymorphisms in peroxisome proliferator-activated receptor γ and their association with plasma levels of resistin and the metabolic syndrome in a South Indian population. J Biosci.

[15] Li Q, Chen R, Bie L, Zhao D, Huang C, Hong J (2015). Association of the variants in the PPARG gene and serum lipid levels: a meta-analysis of 74 studies. J Cell Mol Med.

[16] Sjöholm K, Pajunen P, Jacobson P, Karason K, Sjöström CD, Torgerson J (2015). Incidence and remission of type 2 diabetes in relation to degree of obesity at baseline and 2 year weight change: the Swedish Obese Subjects (SOS) study. Diabetologia..

[17] Cederberg H, Stančáková A, Yaluri N, Modi S, Kuusisto J, Laakso M (2015). Increased risk of diabetes with statin treatment is associated with impaired insulin sensitivity and insulin secretion: a 6 year follow-up study of the METSIM cohort. Diabetologia..

[18] Rooki H, Haerian M-S, Azimzadeh P, Ebrahimi M, Mirhafez R, Ferns G (2013). Distribution and genotype frequency of the C1431T and pro12ala polymorphisms of the peroxisome proliferator activator receptor gamma gene in an Iranian population. Indian J Human Genet.

[19] Cole S, Mitchell B, Hsueh W, Pineda P, Beamer B, Shuldiner A (2000). The Pro12Ala variant of peroxisome proliferator-activated receptor-γ2 (PPAR-γ2) is associated with measures of obesity in Mexican Americans. Int J Obes Relat Metab Disord.

[20] Fornage M, Jacobs DR, Steffes MW, Gross MD, Bray MS, Schreiner PJ (2005). Inverse effects of the PPARγ2 Pro12Ala polymorphism on measures of adiposity over 15 years in African Americans and whites. The CARDIA study. Metabolism..

[21] Tai ES, Corella D, Deurenberg-Yap M, Adiconis X, Chew SK, Tan CE (2004). Differential effects of the C1431T and Pro12Ala PPARγ gene variants on plasma lipids and diabetes risk in an Asian population. J Lipid Res.

[22] Du J, Shi H, Lu Y, Du W, Cao Y, Li Q (2012). Tagging single nucleotide polymorphisms in the PPAR-γ and RXR-α gene and type 2 diabetes risk: a case-control study of a Chinese Han population. J Biomed Res.

[23] Shi H, Lu Y, Du J, Du W, Ye X, Yu X (2012). Application of back propagation artificial neural network on genetic variants in adiponectin ADIPOQ, peroxisome proliferator-activated receptor-γ, and retinoid X receptor-α genes and type 2 diabetes risk in a Chinese Han population. Diabetes Technol Ther.

[24] Lamri A, Khalil CA, Jaziri R, Velho G, Lantieri O, Froguel P (2012). Dietary fat intake and polymorphisms at the PPARG locus modulate BMI and type 2 diabetes risk in the DESIR prospective study. Int J Obes.

[25] Mohamed MBH, Mtiraoui N, Ezzidi I, Chaieb M, Mahjoub T, Almawi W (2007). Association of the peroxisome proliferator-activated receptor-γ2 Pro12Ala but not the C1431T gene variants with lower body mass index in type 2 diabetes. J Endocrinol Invest.

[26] Tai ES, Sim XL, Ong TH, Wong TY, Saw SM, Aung T (2009). Polymorphisms at newly identified lipid-associated loci are associated with blood lipids and cardiovascular disease in an Asian Malay population. J Lipid Res.

[27] Willer CJ, Schmidt EM, Sengupta S, Peloso GM, Gustafsson S, Kanoni S (2013). Discovery and refinement of loci associated with lipid levels. Nat Genet.

[28] Chang MH, Yesupriya A, Ned RM, Mueller PW, Dowling NF (2010). Genetic variants associated with fasting blood lipids in the U.S. population: Third National Health and Nutrition Examination Survey. BMC Med Genet.

[29] Do R, Willer CJ, Schmidt EM, Sengupta S, Gao C, Peloso GM (2013). Common variants associated with plasma triglycerides and risk for coronary artery disease. Nat Genet.

[30] Holmes MV, Asselbergs FW, Palmer TM, Drenos F, Lanktree MB, Nelson CP (2014). Mendelian randomization of blood lipids for coronary heart disease. Eur Heart J..

[31] Saleheen D, Soranzo N, Rasheed A, Scharnagl H, Gwilliam R, Alexander M (2010). Genetic determinants of major blood lipids in Pakistanis compared with Europeans. Circ Cardiovasc Genet..

[32] Kim YJ, Go MJ, Hu C, Hong CB, Kim YK, Lee JY (2011). Large-scale genome-wide association studies in East Asians identify new genetic loci influencing metabolic traits. Nat Genet.

[33] Ordovas JM, Robertson R, Cléirigh EN (2011). Gene–gene and gene–environment interactions defining lipid-related traits. Curr Opin Lipidol.

[34] Adeyemo A, Rotimi C (2009). Genetic variants associated with complex human diseases show wide variation across multiple populations. Public Health Genomics..

[35] Dumitrescu L, Carty CL, Taylor K, Schumacher FR, Hindorff LA, Ambite JL (2011). Genetic determinants of lipid traits in diverse populations from the population architecture using genomics and epidemiology (PAGE) study. PLoS Genet.

[36] Chang M-h, Ned RM, Hong Y, Yesupriya A, Yang Q, Liu T (2011). Racial/ethnic variation in the association of lipid-related genetic variants with blood lipids in the US adult population. Circulation: Cardiovascular. Genetics.

